# Repurposing an In Vitro Measles Virus Dissemination Assay for Screening of Antiviral Compounds

**DOI:** 10.3390/v14061186

**Published:** 2022-05-29

**Authors:** Katharina S. Schmitz, Mona V. Lange, Lennert Gommers, Kim Handrejk, Danielle P. Porter, Christopher A. Alabi, Anne Moscona, Matteo Porotto, Rory D. de Vries, Rik L. de Swart

**Affiliations:** 1 Department of Viroscience, Erasmus MC, 3015 GD Rotterdam, The Netherlands; k.schmitz@erasmusmc.nl (K.S.S.); mona.lange@pei.de (M.V.L.); l.gommers@erasmusmc.nl (L.G.); k.handrejk@erasmusmc.nl (K.H.); r.d.devries@erasmusmc.nl (R.D.d.V.); 2 Gilead Sciences, Foster City, CA 94404, USA; danielle.porter@gilead.com; 3 Robert Frederick Smith School of Chemical and Biomolecular Engineering, Cornell University, Ithaca, NY 14850, USA; caa238@cornell.edu; 4 Department of Pediatrics, Columbia University Medical Center, New York, NY 10032, USA; am939@cumc.columbia.edu (A.M.); mp3509@cumc.columbia.edu (M.P.); 5 Center for Host–Pathogen Interaction, Columbia University Irving Medical Center, New York, NY 10032, USA; 6 Department of Physiology and Cellular Biophysics, Columbia University Irving Medical Center, New York, NY 10032, USA; 7 Department of Microbiology and Immunology, Columbia University Irving Medical Center, New York, NY 10032, USA; 8 Department of Experimental Medicine, University of Campania “Luigi Vanvitelli”, 81100 Caserta, Italy

**Keywords:** measles, measles virus, antiviral, antiviral assay, remdesivir, fusion inhibitory peptide, pandemic response box

## Abstract

Measles virus (MV) is a highly contagious respiratory virus responsible for outbreaks associated with significant morbidity and mortality among children and young adults. Although safe and effective measles vaccines are available, the COVID-19 pandemic has resulted in vaccination coverage gaps that may lead to the resurgence of measles when restrictions are lifted. This puts individuals who cannot be vaccinated, such as young infants and immunocompromised individuals, at risk. Therapeutic interventions are complicated by the long incubation time of measles, resulting in a narrow treatment window. At present, the only available WHO-advised option is treatment with intravenous immunoglobulins, although this is not approved as standard of care. Antivirals against measles may contribute to intervention strategies to limit the impact of future outbreaks. Here, we review previously described antivirals and antiviral assays, evaluate the antiviral efficacy of a number of compounds to inhibit MV dissemination in vitro, and discuss potential application in specific target populations. We conclude that broadly reactive antivirals could strengthen existing intervention strategies to limit the impact of measles outbreaks.

## 1. Introduction

Measles virus (MV) is a highly contagious respiratory virus that causes outbreaks associated with significant morbidity and mortality. Multiple initiatives, including the Measles and Rubella Initiative, set out to eliminate measles [1]. Safe and effective live-attenuated measles vaccines are available, but vaccination hesitancy and vaccination coverage gaps have decreased MV-specific herd immunity, leading to global resurgences of measles. Yearly, measles accounts for more than 100,000 deaths, mostly in children under the age of five in Africa and Asia [2]. In recent years, increasing numbers of measles cases and associated deaths were recorded. In 2019, measles cases hit their highest number in 23 years and were responsible for more than 207,000 deaths [3]. Case numbers drastically declined in 2020 and 2021 due to social distancing measures and travel restrictions, but a further measles surge is expected in the upcoming years as measles immunization programs have been affected by the COVID-19 pandemic [4,5].

### 1.1. Measles Virus

MV is a member of the genus *Morbillivirus* of the family *Paramyxoviridae* and has a negative-sense unsegmented RNA genome [6]. MV is a monotypic virus. Although eight different clades and 24 genotypes have been identified, most of these no longer circulate, and genotype B3 and D8 are currently the most prevalent worldwide, while genotype H1 still circulates in China [7,8]. Despite harboring an RNA-dependent RNA-polymerase (RdRp) that lacks proofreading capacity, the mutation rate of MV is low. Most likely, the virus has a narrow fitness peak and is limited in the mutations it can tolerate. Therefore, vaccines developed in the 1960s still offer clinical protection from measles. The MV genome encodes six structural and two non-structural proteins [9]: The nucleoprotein (N) encapsidates the viral genome. The two viral transmembrane glycoproteins, the hemagglutinin (H) and the fusion protein (F), are responsible for cell attachment and entry [10,11]. Entry is initiated by the H protein, binding to the cellular receptors CD150 (SLAM) or nectin-4 [12,13,14] and triggering confirmational changes in the F protein. These conformational changes facilitate the fusion of the viral and cell membrane. Subsequently, the genome content is released into the cell, transcribed by the viral RdRp (large protein, L) and its cofactor phosphoprotein (P), and by orchestration of the matrix (M) protein, all viral proteins are ultimately assembled at the cell membrane before being released [15,16]. Detailed characterization of the two non-structural proteins V and C, which are transcribed from the P gene, remains to be performed, but both appear to be virulence factors involved in antagonizing innate immune responses [17].

### 1.2. Pathogenesis

Measles virus is highly contagious and has a basic reproductive number (R_0_) of 12 – 18 [18]. It enters via the respiratory tract, infects CD11c^+^ myeloid cells, probably alveolar macrophages and DCs, which disseminate the virus to the local lymphoid tissues [19,20]. In the draining lymph nodes, MV infects CD150^+^ B- and T-lymphocytes, which effectively amplify and distribute the virus throughout the body [21]. Ultimately, a major fraction of CD150^+^ white blood cells, including lymphocytes and DCs in the epithelial submucosa, are infected. Those infected cells can transfer the virus to nectin-4-positive respiratory epithelial cells from where MV is released via the upper respiratory tract and transmitted by aerosols or small droplets [22,23].

The MV incubation phase is between 9 and 19 days, followed by a prodromal phase, which is characterized by fever, conjunctivitis, malaise, cough and coryza. The preferential massive infection and depletion of CD150-positive memory lymphocytes results in transient immune amnesia [24], which may last more than two years after measles. This leaves measles patients susceptible to opportunistic infections and leads to increased mortality due to infectious complications [25]. Paradoxically, a robust MV-specific immune response is formed, which clears the infection and lasts for life [26]. The typical measles rash following the prodromal phase with a two-to-four-day delay is a result of MV-specific T-cell invasion into the skin to clear infected keratinocytes [27].

In rare cases, measles may also lead to neurological complications such as acute post measles encephalitis (APME), measles inclusion body encephalitis (MIBE) or subacute sclerosing panencephalitis (SSPE). While APME is associated with an auto-immune reaction against oligodendrocytes, fatal in 20% of the cases, MIBE and SSPE occur after MV infection of the central nervous system (CNS) in immunocompromised or immunocompetent patients, respectively. Both MIBE and SSPE are almost always fatal and can only be treated symptomatically (reviewed in [28]).

### 1.3. Treatment Challenges

Given its high transmissibility, herd immunity levels of 95% are required for sufficient measles control [29]. Decreasing vaccination coverage results in rising case numbers and more complications associated with measles. Therefore, there is a significant risk for individuals who cannot be vaccinated, such as young infants (<6 months) and immunocompromised individuals but also for unvaccinated healthcare workers and their vulnerable patients. Antivirals could be used for measles prophylaxis in an insufficiently protected population or serve as treatment during acute infection or measles-associated long-term complications. However, to date, no specific antiviral treatment for MV is approved. The pathogenesis of measles is complex, making antiviral treatment challenging. Measles has a long incubation time, and initial symptoms are often attributed to other infectious diseases, partly because general practitioners are rarely confronted with measles. Rather than staying confined to the respiratory tract, MV rapidly spreads systemically, even before the onset of symptoms. Once in the dissemination phase, it is likely that treatment will have limited impact on disease outcome or immune suppression, leaving an extremely narrow treatment window in the extended incubation phase to prevent lymphopenia.

On the other hand, measles outbreaks usually occur in geographically clustered communities. Rapid identification of index cases allows for contact tracing of unvaccinated individuals and early post-exposure treatment. Post-exposure vaccination or passive immunization with intra-venous immunoglobulin (IVIg) is recommended by the World Health Organization (WHO) and has been used with diverse levels of success [30,31,32]. Effective measles prevention and treatment requires optimal infrastructure and resources, which are not always present in countries where measles is most prevalent. 

### 1.4. In Vitro Testing

Antiviral compounds investigated for in vitro activity against MV include nucleoside analogs, non-nucleoside polymerase inhibitors, fusion and entry inhibitors, host-directed compounds, plant extracts, disinfectants, anti-microbial agents, and interferons (Table 1). 

Nucleoside analogs commonly target viral replication, particularly the viral DNA or RNA polymerase. They contain non-canonical bases that are integrated into the viral genome after intracellular phosphorylation [89,90]. The purine-analog ribavirin has demonstrated broad spectrum antiviral activity [91] and has been approved to treat several viruses, including respiratory syncytial virus (RSV) [49]. Multiple mechanisms of actions are being discussed, including the inhibition of viral polymerase and viral RNA capping [92]. Ribavirin base-pairs with equal efficiency with cytosine and uracil [93], resulting in hypermutations of the newly synthesized strand, which block virus replication via error catastrophe [94]. However, resembling adenosine and guanosine also carries the disadvantage of increased interaction with the host cell machinery, leading to poor selectivity and toxicity, which may result in adverse events such as anemia [92]. 

Remdesivir (GS-5734) is a nucleoside analog prodrug with broad-spectrum in vitro antiviral activity against RNA-viruses from many virus families [41]. As an adenosine-analog, remdesivir interferes with the viral RNA synthesis [95] and leads to delayed RNA-chain termination three to five nucleotides after its incorporation [92,96]. Remdesivir has demonstrated efficacy in the treatment of both hospitalized and non-hospitalized COVID-19 patients in multiple clinical trials and was initially approved by the U.S. Food and Drug administration (FDA) in 2020 [97]. 

The RdRp presents a promising target for broad-spectrum non-nucleoside antiviral drugs, as it is highly conserved across several RNA viruses such as influenza viruses, coronaviruses and paramyxoviruses, including MV [98]. Different non-nucleoside inhibitors such as ERDRP-0519 or AS-136a show efficacy in in vitro and in vivo studies [48,51]. Targeting the L subunit of the RdRp, ERDRP-0519 inhibits the formation of phosphodiester bonds during initiation of RNA synthesis and RNA elongation [51].

Next to the viral polymerase, an important protein in the MV lifecycle is the fusion protein. The fusion of enveloped viruses with the host cell is a key step in their infectivity, and interference with this process can lead to highly effective antivirals. Fusion inhibitors for class I viral fusion proteins are often peptides or small molecules and can have multiple mechanisms of action. Fusion inhibitory peptide (FIP) was first used as an acronym for Z-D- Phe-L-Phe-L-Gly, a synthesized oligopeptides with an amino acid sequence that is similar to the N-terminal region of F1 [54]. FIP-analogs block membrane fusion by stabilizing the prefusion state of the viral F protein. In contrast, peptides mimicking one of the two heptad repeat (HR) domains capture the viral F protein in a post-triggering state and freeze the fusion process at the beginning.

Overall, a dozen different antiviral assays with minor modifications have been performed to evaluate the antiviral potency of numerous antiviral drugs against MV (Table 2).

Given the pathogenesis of measles, highly predictive in vitro models mimicking the in vivo situation are needed. Considering the earliest realistic timepoint of treatment and the early events during MV infection, an in vitro assay resembling the dissemination phase of MV in immune cells is anticipated to provide translational value. Moreover, wild-type-based viruses in comparison to vaccine-based viruses seem superior when evaluating the antiviral potential of a drug to clinically relevant MV. Therefore, we repurposed a previously established MV-dissemination assay in lymphoid cells for screening of antivirals [100]. In this assay, we evaluated the antiviral efficacy of IVIg, remdesivir and a selection of fusion inhibitory lipopeptides designed to inhibit MV fusion in comparison to ribavirin. Moreover, we screened 153 antiviral candidates from the Medicines for Malaria Venture Pandemic Response Box for their potential to inhibit MV dissemination.

## 2. Materials and Methods

### 2.1. Cells and Viruses 

All experiments were performed in human Epstein–Barr-virus-transformed (EBV) B-lymphoblastic cell lines (B-LCL). B-LCLs were maintained in RPMI-1640 medium (Lonza) supplemented with penicillin (1000 U/mL), streptomycin (100 µg/mL), 2 mM L-glutamine and 10% fetal bovine serum (FBS). Virus stocks were grown in B-LCL, MRC-5 cells or Vero-human-SLAM (VHS) cells. MRC-5 and VHS were maintained in DMEM (Lonza) supplemented with penicillin, streptomycin, L-glutamine and 10% FBS. The recombinant MV expressing the reporter protein Venus at position three of the genome (rMV^KS^Venus(3)) was based on a clinical isolate from Khartoum, Sudan [101]; rMV^KS^Venus(3) was grown in B-LCL. The recombinant MV expressing the reporter protein EGFP at position three of the genome (rMV^EZ^EGFP(3)) was based on the Edmonston-Zagreb vaccine virus [102]; rMV^EZ^EGFP(3) was grown in MRC-5 cells. All clinical isolates (MVi/Khartoum.SUD/34.97/2 − genotype B3, MVi/Bilthoven. NLD/1991 − genotype C2, MVi/Amsterdam.NLD/19.11 − genotype D4, MVi/ Dodewaaard.NLD/29.13 − genotype D8, MVi/Amsterdam.NLD/49.97 − genotype G2, MVi/Amsterdam/NLD/27.97 − genotype H1) used in this study were isolated in B-LCLs, and stocks were grown in VHS. 

### 2.2. Antiviral Compounds

IVIg (Privigen) was diluted to 100 mg/mL and used at final concentrations of 2 mg/mL to 0.2 ng/mL (10-fold dilutions). Remdesivir was obtained from Gilead Sciences, Inc. (Foster City, CA, USA) as a 20 mM solution dissolved in DMSO. In all assays, remdesivir was used at final concentrations of 10 µM to 0.1 nM (5-fold dilution series). Ribavirin was diluted in DMSO to a concentration of 25 mM and used at final concentrations of 250 µM to 2 µM (2-fold dilution series). All fusion inhibitory peptides were received as a 5 mM solution and used at final concentrations of 10 µM to 0.1 nM (5-fold dilution series). The Pandemic Response Box was designed and kindly supplied by Medicines for Malaria Venture (MMV). Compounds classified as ‘antiviral’ were diluted to 2 mM in DMSO and aliquoted until further use. For primary screenings, antivirals were tested at 20 µM and 4 µM. Potential hits were defined as compounds that resulted in less than 50% MV infection at a concentration of 20 μM and/or less than 90% MV infection at a concentration of 4 μM, independent of toxicity and time point of evaluation (48 or 72 h post co-culture, hpc). Those hits were re-screened at 4 µM, 800 nM, 160 nM and 32 nM. 

### 2.3. In Vitro MV Dissemination Assay

**Figure 1:**
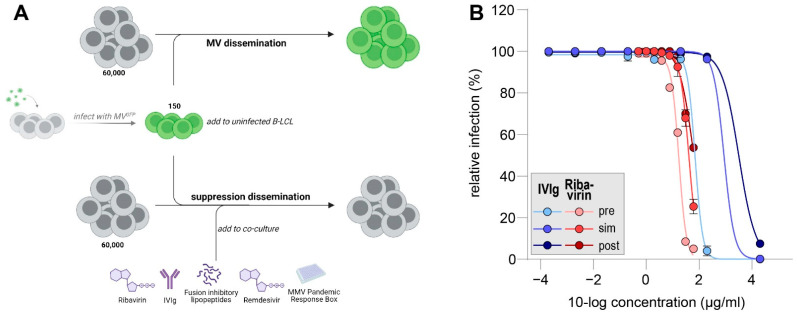
(**A**) Schematic representation of antiviral dissemination assay. Infected cells are presented in green. (**B**) Dose–response curves for IVIg (blue) and ribavirin (red) in MV dissemination assay. Addition of inhibitors was six hours pre co-culture (pre, light shading), at the moment of co-culture (sim (simultaneous), medium shading) or six hours post co-culture (post, dark shading). Infection percentages were normalized to untreated cultures and errors are depicting the SEM.

A previously established MV-dissemination assay was used to test the efficacy of the selected antiviral compounds (Figure 1a) [100]. B-LCLs were infected with MV (clinical isolate or recombinant MV) for 48 to 72 h. Infection percentage in the live cell fraction was determined using flow cytometry (BD FACS Lyric), either by measuring expression of the green fluorescent reporter protein or by staining for MV nuclear protein (NP). In the assay, 150 MV-infected cells were added to 60,000 uninfected B-LCLs. Antiviral compounds were added to those cultures simultaneously, six hours before or six hours after co-culture. As control, co-cultures were performed without antiviral (in the presence of equimolar concentrations of DMSO). MV dissemination and compound or vehicle toxicity were analyzed 48 and 72 h post co-culture. For toxicity, cell viability was evaluated based on FSC/SSC and for infection percentage FITC^+^ events were recorded. In assays performed with clinical isolates, cells were washed in PBS, fixed and permeabilized (Cytofix-Cytoperm, BD) according to the manufacturer’s instructions and stained with anti-MV-NP/FITC (KK2, Millipore). Data shown were collected 48 h post co-culture unless differently stated. At this point, infection levels in control co-cultures were >90%. Remdesivir, ribavirin and fusion-inhibitory peptides were tested in duplicate in three or more independent assays.

### 2.4. Data Analysis

The half-maximal inhibitory concentration (IC_50_) was determined using a four-parameter non-linear regression on normalized (to DMSO-treated co-cultures) and transformed infection percentages. All analyses were performed in GraphPad Prism 9.

## 3. Results

### 3.1. IVIg Inhibitis MV-Dissemination at Physiological Concentrations

We designed an MV-dissemination assay to evaluate the efficacy of viral inhibitors in the dissemination phase. The assay assesses the cell-to-cell spread of MV in the presence or absence of antiviral compounds. As MV replication in B-LCLs requires approximately 24 h, a window of post-exposure treatment is given to address multiple treatment conditions (Figure 1A).

We validated our assay using multiple compounds which do not inhibit MV and compared those to ribavirin, often shown to inhibit MV in vitro (data not shown). 

After successful validation, we were aiming to evaluate the potency of IVIg to inhibit wild-type MV dissemination and compare it to ribavirin. To this end, we used a recombinant MV based on a clinical isolate from Khartoum, Sudan (Genotype B3), which expresses a fluorescent protein at position three of its genome (rMV^KS^Venus(3)). Serial dilutions of IVIg or ribavirin were added either before, simultaneously with or after the addition of MV-infected cells to uninfected cultures and incubated for 48 h. Next, Venus-expressing cells were detected by flow cytometry as a measure of MV infection (Figure 1B). 

In all treatment conditions, the highest tested concentration of IVIg (2 mg/mL) inhibited MV dissemination almost completely. MV dissemination was also suppressed when cells were incubated with 0.2 mg/mL of IVIg prior to the addition of MV-infected B-LCLs. The further dilution of IVIg led to MV infection of almost all uninfected cells in all treatment conditions. The half-maximal inhibitory concentrations (IC_50_) were 0.07 mg/mL, 0.87 mg/mL and 3.2 mg/mL for pre-incubation, simultaneous incubation or post-exposure incubation, respectively. Ribavirin only effectively inhibited MV-dissemination when pre-incubated with uninfected cells. If ribavirin was added simultaneously or six hours post-co-culture, infection percentages at 61 µg/mL were 25% or 54%, respectively. IC_50_ values for ribavirin pre-incubation were 17 µg/mL, for ribavirin incubation at the start of co-culture 40 µg/mL and for ribavirin post-exposure treatment 62 µg/mL. Our data support an inhibitory effect of IVIg and ribavirin on the dissemination of MV. Importantly, this effect is highly time-dependent, leading to reduced antiviral efficacy when inhibitors are added post-exposure.

### 3.2. Remdesivir Efficiently Inhibitis MV-Dissemination at Sub-Micromolar Concentrations

**Figure 2:**
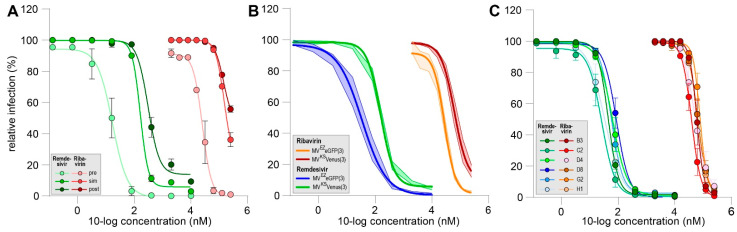
(**A**) Representative dose–response curves for remdesivir (green) and ribavirin (red) in the MV dissemination assay. Addition of compounds was six hours pre-co-culture (pre, light shading), simultaneous with start of co-culture (sim, medium shading) or six hours post co-culture (post, dark shading). (**B**) Mean dose–response curves for remdesivir (blue and green) and ribavirin (orange and red) in MV dissemination assay comparing vaccine-strain-based (blue and orange) and wild-type-based (green and red) viruses (*n* ≥ 5). (**C**) Mean dose–response curves for remdesivir (green to blue) and ribavirin (red to orange) in MV dissemination assay comparing different clinical isolates (*n* = 2). (**A**–**C**) Infection percentages were normalized to untreated cultures and errors are depicting the SEM.

Next, we evaluated the effect of remdesivir on rMV^KS^Venus(3)-dissemination in pre-, simultaneous or post-co-culture treatment regimens and compared this to ribavirin (Figure 2A). Remdesivir inhibited its dissemination in all tested conditions. The pre-exposure of uninfected cultures with remdesivir resulted in almost complete inhibition of dissemination at 80 nM or higher. If remdesivir was added to the co-culture simultaneously or six hours post co-culture, 10% infection or less was measured at 400 nM and 10 µM, respectively. Mean IC_50_ values of four or more experiments were 54 nM for pre-addition, 175 nM for simultaneous addition and 304 nM for post-addition conditions. Similar differences as those related to the timing of treatment were observed for ribavirin, as shown in Figure 1B. The mean IC_50_ values for ribavirin obtained in three or more experiments were 38 µM, 72 µM and 119 µM for pre-, simultaneous or post-addition, respectively. Notably, IC_50_ values for post co-culture addition varied greatly between assays, and no upper limit of a 95% confidence interval could be determined. 

Next, we assessed the influence of simultaneous addition of remdesivir and ribavirin on a vaccine strain-based (rMV^EZ^EGFP(3)) or wildtype-based (rMV^KS^Venus(3)) recombinant MV (Figure 2B). rMV^EZ^EGFP(3) dissemination was slightly better inhibited by remdesivir and ribavirin when compared to the rMV^KS^Venus(3). The corresponding mean IC_50_s were 34 nM and 156 nM (4.5-fold decrease) for remdesivir and 28 µM and 63 µM for ribavirin, respectively.

Finally, we tested the efficacy of remdesivir and ribavirin added simultaneously against low-passage clinical MV isolates belonging to different genotypes (Figure 2C). A total of six different virus isolates were evaluated and no inter-genotype differences were detected. IC_50_ values for remdesivir varied between 27 nM and 87 nM and for ribavirin between 39 µM and 77 µM. Generally, IC_50_ values for all wild-type viruses appeared slightly lower when compared to the recombinant wild-type-based virus. This could be possibly attributed to the method of infection detection as recombinant wild-type-based virus infections were evaluated based on EGFP or Venus expression, while the N protein was stained for all wild-type viruses. 

In conclusion, the nucleoside-analog remdesivir inhibited MV dissemination at sub-micromolar concentrations.

### 3.3. Fusion Inhibitory Lipopeptides Prevent MV Dissemination at Nanomolar Concentrations

**Figure 3:**
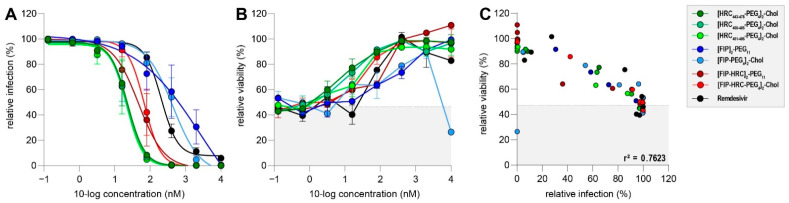
(**A**) Dose–response curves of three different lipopeptide classes in MV dissemination assay. Peptides depicted in green represent HRC-based lipopeptides, peptides in blue represent FIP-based lipopeptides and peptides in red represent HRC–FIP-hybrid-based peptides. Remdesivir (black) was used as a control. Infection percentages were normalized to untreated co-cultures, and errors are depicting the SEM. (**B**) Relative viability of co-cultures treated with different lipopeptides and infected with rMV^KS^Venus(3). Shaded background depicts mean viability in infected but untreated co-cultures. Error bars depict the SEM. (**C**) Correlation of infection percentage and viability in infected and treated co-cultures. Shaded background depicts mean viability in infected but untreated co-cultures.

We evaluated three classes of previously described fusion inhibitory lipopeptides in our MV dissemination assay (Figure 3A). Peptides mimicking different sequences of the C-terminal heptad repeat domain (HRC) of the fusion protein (green) [60,61] inhibited rMV^KS^Venus(3) dissemination completely at 80 nM or higher. FIP ((Z)-d-Phe-l-Phe-Gly)-modified lipopeptides [54,64] (blue) performed less well, and FIP-HRC hybrid lipopeptides [64] (red) had an intermediate effect on viral dissemination when compared to HRC- or FIP-based peptides. IC_50_ values ranged from 22 nM for HRC-based peptides, to 47–68 nM for hybrid peptides and more than 400 nM for FIP-based peptides.

Peptide cytotoxicity was determined using flow cytometry. With the exception of the [FIP-PEG_4_]_2_-chol peptide (light blue), no lipopeptide was cytotoxic at the highest tested concentration (Figure 3B). Cellular viability was inversely proportional to infection percentages (r^2^ = 0.76) suggesting that cytotoxicity was induced by viral infection, rather than drug-related (Figure 3C). 

Overall, these data demonstrate that fusion inhibitory peptides can efficiently inhibit MV dissemination at low concentrations with neglectable cytotoxicity. Importantly, the different classes of fusion inhibitors varied in efficacy, with HRC-based lipopeptides performing best in the dissemination assay.

### 3.4. MMV Pandemic Response Box Screening

**Figure 4:**
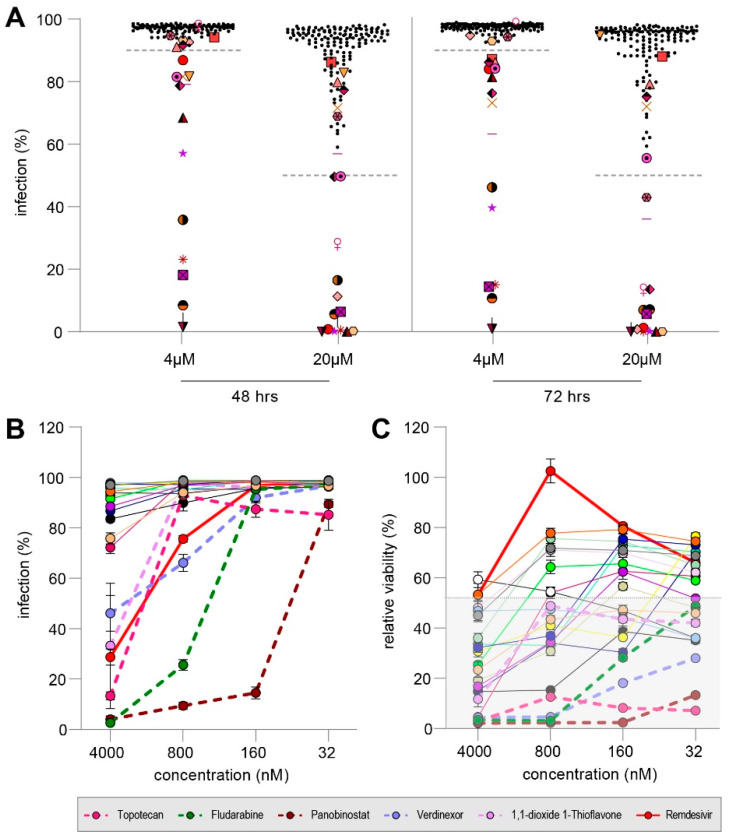
(**A**) Infection percentage reached in co-cultures treated with 160 diverse compounds at 4 µM or 20 µM evaluated at 48 hpc (left) or 72 hpc (right). Dotted lines indicate cut-off to qualify for re-screening. Overall, 21 compounds reduced MV dissemination (enlarged colored symbols, one symbol per compound). (**B**) Rescreening of 21 compounds and remdesivir for their inhibitory effect on MV dissemination. Dotted lines indicate compounds regarded effective. Error bars depict the SEM. (**C**) Relative viability of all 21 compounds and remdesivir. Dotted lines indicate compounds which were regarded effective, but proof to be cytotoxic. Shaded background depicts mean viability in infected but untreated co-culture.

The Pandemic Response Box (PRB) contains 400 diverse molecules of antiviral, antibacterial or antifungal properties. Selected compounds are at various stages of research and development and have been characterized into the three categories based on literature. Initially, we screened all 153 compounds categorized as antivirals, 5 antibacterials and 2 antifungals for their inhibitory properties against rMV^KS^Venus(3) at two concentrations in our dissemination assay. Hits were defined as compounds that resulted in less than 50% MV infection at a concentration of 20 µM and/or less than 90% MV infection at a concentration of 4 µM, independent of toxicity and the time point of evaluation (48 or 72 h post-co-culture (hpc)). Importantly, using those cut-off criteria, we overestimated the number of compounds with possible anti-MV effect. At 48 hpc, 14 compounds showed less than 50% MV infection at a concentration of 20 μM, while this was measured for 15 compounds at 72 hpc. Using compounds at a concentration of 4 μM, 14 and 16 compounds exhibited less than 90% MV infection 48 or 72 hpc, respectively. In total, 21 compounds reduced the percentage of MV-infected cells and qualified for further testing (Figure 4A, enlarged, colored symbols, one symbol per compound).

We used a five-fold dilution series starting from 4 µM to test the 21 compounds, namely, MMV394033, Emricasan, MMV1782101, MMV1782115, Ozanimod, MMV690621, MMV642550, Topotecan, Panobinostat, Selinexor, URMC-099-C, Fludarabine Triapine, DNDI1417411, MMV1782214, MMV218827, Rubitecan, ML324, Verdinexor, MMV019724 and 1,1-dioxide 1-Thioflavone, with remdesivir as a control. Five compounds (Topotecan, Fludarabine, Panobinostat, Verdinexor and 1,1-dioxide 1-Thioflavone) inhibited MV dissemination by more than 50% at 4 µM. (Figure 4B, dotted line). However, all five compounds proved to be cytotoxic with a relative viability of less than 20% in co-cultures infected and treated with the compounds (Figure 4C). Therefore, we concluded that no antiviral compound of the PRB was able to inhibit MV dissemination efficiently but that the induced cytotoxicity led to decreased infection.

## 4. Discussion

We have repurposed an MV dissemination assay to test the efficacy of various antiviral compounds. Originally, a low number of infected EBV-transformed human B-lymphoblastic cells (B-LCL) were co-cultured with a large number of uninfected cells and MV-specific, HLA-matched T-cell clones (TCC), which suppressed the dissemination of MV. The repurposed assay uses B-LCLs as target cells to mimic the early dissemination of MV in lymphocytes. B-LCL express two MV receptors, CD150 and CD46. While CD46 is no natural MV receptor and can only be used by MV vaccine strains or cell culture adapted strains, CD150 is expressed on myeloid and lymphoid cells and used by MV vaccine strains and wild-type strains. However, non-human primate studies have shown that intra-muscular injected MV vaccine strains preferentially target CD150-expressing DCs and macrophages instead of CD46-expressing myocytes and therefore resemble the tropism of wild-type MV viruses [102]. Studies with wild-type-based MV in NHPs have shown that MV amplification starts in bronchus-associated lymphoid tissues [19] or local draining lymph nodes [20], which is represented by a small amount of infected B-LCLs in a large number of uninfected cells in our antiviral assay. Local amplification leads to dissemination throughout the entire body. In humans, peripheral blood mononuclear cells (PBMC) are infected as early as five days before the onset of rash, and the peak of the cell-associated viremia is measured before the onset of rash [103]. In NHPs, viremia peaks at nine days post-inoculation and also precedes the onset of rash [24], suggesting that the NHP model closely resembles human disease. Simultaneously, the lowest white blood cell count can also be measured during or just after peak viremia, which is associated with follicular depletion in lymph nodes [24]. This indicates progressive lymphodepletion, both in peripheral blood and in lymphoid tissues. In our in vitro antiviral assay, we achieved best treatment outcomes if antivirals were added prior to or simultaneously with the start of MV dissemination. Treatment of measles patients should therefore ideally be initiated before the start of viremia, i.e., more than five days before the onset of rash, as this marks the highest chance of a mostly local MV infection

IVIg and measles vaccination are the only currently approved post-exposure intervention methods. Usually, IVIg is administered within six days after exposure and is often limited to immunocompromised contacts, pregnant women and children below the age of 6 months. Dependent on the country, dosages vary between 150 mg/kg bodyweight and 400 mg/kg bodyweight leading to maximum concentrations in blood of 3–8 mg/mL (assuming approx. 50 mL serum per kg bodyweight) [104]. In our assay, IC_50_ values, but not complete inhibition, for six-hour post-exposure treatment with IVIg were 3.2 mg/mL, which is similar to what is achieved in vivo and what possibly explains the variable efficacy in patients. Importantly, in our assay, IVIg effectivity increased with earlier treatment, supporting the importance of early treatment in vivo [31,32,105]. 

We have repeatedly shown that remdesivir efficiently inhibits MV dissemination at half-maximal inhibitory concentrations below 180 nM, regardless if wild-type-based viruses or vaccine-strain-based viruses were used. In line with this, Lo et al. estimated the IC_50_ of remdesivir against MV infection in HeLa cells at 37 nM, in VeroE6 cells at 5 µM, in NCl-H358 cells at 25 nM and in HSAEC1-KT cells at 63 nM, using an rMV^EZ^EGFP(3) reporter-based assay [41,42]. Our assay resembles the early dissemination phase and mimics in vivo cell-to-cell viral spread instead of cell-free virus production. Similar to the time-dependent effect seen for IVIg, remdesivir inhibited viral dissemination best when administered early. However, almost complete inhibition could also be observed when co-cultures were treated 6 h post-initiation of co-culture and likely all uninfected cells were already exposed to MV. It is unclear how those in vitro results will translate in vivo and whether remdesivir can be established as an effective post-exposure prophylaxis or treatment for measles. Crucial considerations include the emergence of resistance mutations which remain to be investigated, the timing of treatment initiation and the translation of the in vitro determined effective dose to an in vivo administered dose, reaching high enough systemic concentrations to inhibit MV in all target organs. 

Remdesivir’s intravenous administration is feasible for the treatment of severe measles cases or neurological complications which require hospitalization, but makes it less attractive for early post-exposure prophylaxis. Therefore, advances to design an orally available compound that generates the same bioactive metabolite as remdesivir need to be made. 1-O-octadecyl-2-O-benzyl-sn-glycerylester (ODBG) lipid-modified monophosphate prodrug (ODBG-P-RVn) is an orally available derivative of Nuc, remdesivir’s parent nucleotide, and proved to be stable in plasma and highly active against MV (82–160 nM) [42], potentially making it more widely available and easier accessible. 

Intranasal administration of fusion inhibitory peptides has been suggested as prophylaxis for MV and SARS-CoV-2, preventing virus infection in the respiratory tract [61,106]. However, treatment of measles, as a primarily systemic disease, may need to be looked at from the perspective of other systemic infections such as HIV. For treatment-experienced HIV patients, enfuvirtide (T20), the only marketed fusion inhibitor, can be used but needs to be administered twice daily by subcutaneous injection, possibly challenging patient compliance [107,108]. Moreover, drug resistance to enfuvirtide has been numerously reported in patients [109]. For MV, resistant mutants to FIP and related fusion inhibitors have been detected or were induced by selective pressure in in vitro experiments [56,110]. Fortunately, a fusion inhibitory peptide with an unrelated working mechanism still conferred protection in CD150 transgenic suckling mice infected with FIP-resistant MV [63]. 

Mechanistically, fusion-inhibitory peptides based on one HR-domain directed against viruses containing a class I fusion protein such as MV, SARS-CoV-2 or HIV work similarly. Peptides mimic one of the two HR-domains, which usually facilitate fusion by forming a six-helix bundle (6-HB) with the complementary HR. Increasing concentrations of peptides outcompete the complementation of both HRs, thus inhibiting the transition of the F protein into its post-fusion state. In our MV antiviral assay, we showed the highest antiviral activity for the three peptides derived from HR domains. [HRC_450-485_-PEG_4_]_2_-chol, also referred to as HRC4, has previously been shown to prevent infection in vitro and to reduce viral replication in hippocampal brain slices of neonatal SLAM transgenic mice ex vivo [60,61]. To this end, the investigators had dimerized the HRC peptide and conjugated it to a cholesterol group, as this had been shown previously to enhance antiviral potency [111,112]. 

Next to peptides preventing the transition to post-fusion confirmation, fusion inhibitors stabilizing the prefusion state have been described as early as 1980. FIP, (Z)-d-Phe-l-Phe-Gly, has been investigated for paramyxoviruses and myxoviruses [54]. Lipid-conjugation and dimerization of FIP have once more been shown to increase peptide potency [64]. Importantly, similarly to what has been observed by us, a conjugation of the FIP domain to the HRC domain (hybrid peptide) resulted in a more than 10-fold more potent inhibitor. The authors also report a 10-fold improvement when compared to HRC-based peptides, which is in contrast to our results, in which HRC-based peptides have performed best. While Bovier et al. not only reported a synergistic effect between both peptides, they also speculated that resistance emergence might be less likely considering the dual action mechanism. In our hands, both HRC and hybrid peptides have IC_50_ values below 100 nM and should be further evaluated in post-exposure animal models.

Few antivirals made it to pre-clinical evaluation in MV animal models. The non-human primate model is still considered the gold standard in measles research, as NHPs are the only animal species naturally susceptible to MV infection. Moreover, biodistribution, pharmacokinetics, pharmacodynamics as well as off-target effects of antiviral drugs can be inferred from data obtained in NHPs. However, ethical concerns and high costs led researchers to re-evaluate the use of NHPs as experimental animal model. Rodents are widely being used to determine drug characteristics in vivo. Unfortunately, natural measles cannot be mimicked in rodents, and treatment effects can therefore not be assessed. Alternatively, ferrets can be infected with the closely related morbillivirus canine distemper virus (CDV) and are being used as a surrogate model. In contrast to MV, CDV causes high mortality in ferrets and therefore only mimics a high-susceptibility-with-severe-outcome scenario, rather than a typical MV infection. 

Over the last 10 years the non-nucleoside polymerase inhibitor ERDRP-0519 has been investigated for its anti-MV effect and one of the only antivirals, which has been tested in more than one animal model. Krumm et al. explored the potential of ERDRP-0519 to inhibit CDV in ferrets [50]. They have chosen for a CDV strain which was passaged in ferrets and proved to be highly virulent [113]. All ferrets were treated by gastric gavage with 50 mg/kg for 14 days, starting either 24 h pre-infection or at the onset of viremia (3 days post-infection). Pre-treatment prolonged the survival of ferrets when compared to untreated controls, but ultimately did not prevent fatal outcome. Surprisingly, all ferrets in the post-exposure treatment group survived and only experienced mild lymphopenia. Next, ERDRP-0159 was also tested for its potential as an MV inhibitor in squirrel monkeys infected with a clinical MV isolate from Germany [52]. As measles is usually not lethal in monkeys, the investigators used rash, viral titers in PBMC, viral titers in nose swabs and leukopenia as a measure of efficacy. Next to a pre-exposure-treatment group and an onset-of-viremia-treatment group (3 dpi), they also chose for an onset-of-clinical-signs-treatment group (7 dpi). In all groups, clinical benefits or decreased viral shedding were detected, suggesting a positive influence on the viral epidemiology. However, a clear disease benefit was only seen in the former two groups as treatment 7 dpi did not affect the height of viral load in blood and did also not protect from leukopenia. 

In comparison to ERDRP-0159, multiple fusion inhibitors have been investigated in non-natural hosts for morbilliviruses. The HRC4 peptide and the FIP–HRC hybrid peptide (also used in this study) were evaluated in the cotton rat model, a non-lethal model mostly used to investigate respiratory tract involvement [114]. In all studies, the investigators could show that no virus could be isolated from lungs of intranasally, prophylactically treated and MV-challenged cotton rats [61,62,64]. Moreover, the HRC4 peptide could also be traced in serum and in the brain of cotton rats, suggesting a potential treatment effect after systemic spread.

Characterization of fusion-inhibitory lipopeptides has often been performed in the context of CNS disease, especially SSPE: a late-onset rare but fatal complication of measles. As there is no reliable CNS disease model in a morbillivirus-natural host, most studies have been performed in CD150-transgenic mice, CD150-transgenic suckling mice or CD150 x IFNAR-knock out mice [60,61,62]. Overall, subcutaneous and intranasal application of HRC4 or similar lipopeptides were evaluated in pre-MV-exposure-treatment regimens, showing promising results on survival. Similar to the cotton rat model, the lipopeptides could be detected in the circulation, the lungs as well as the brain with variable concentrations, endorsing some peptides as a potent treatment method, while others could be better used preventative. In other studies, intracranial inoculation of nude mice was used as a model system for MV-induced CNS disease [115]. Watanabe et al. then used an MV-HRC-based peptide as treatment and achieved an increase in survival rate from 0% to 67% [59]. 

CNS disease, specifically SSPE, has further been studied in the hamster model. Studies performed in the 1990s suggested a beneficial effect of ribavirin when administered intracranially at least 36 h post inoculation [116]. In this case, half (5/10) of all treated hamsters survived the lethal infection; full survival was achieved when treatment was initiated as early as 12 h post inoculation with 10 mg/kg/day for 10 days, which correlates to an intra-brain concentration of more than 50 µg/g [117]. The 50% effective dose was calculated to be 1.4 mg/kg/day [116]. Experimental intravenous treatment of two SSPE patients with ribavirin led to neurological improvements [118]. However, both experienced neurological deterioration a few months after the therapy was stopped. Serum concentrations of ribavirin in both patients were between 10 and 20 µg/mL, and CSF concentrations between 7 and 17 µg/mL. As rationalized by the previous hamster studies, prolonged patient improvement could be observed when ribavirin was administered intraventricularly and CSF concentrations were higher than 100 µg/mL [119]. While some studies report in vitro IC_50_ values of ribavirin for MV or the SSPE-strain Yamagata of less than 10 µg/mL [33,34,36,43], we and others determined IC_50_ values of higher than 35 µg/mL [33,73], especially when ribavirin was used as post-exposure treatment. 

Next to efficacy, many other drug characteristics are validated in animal models. A perfect antiviral should have a low toxicity profile and should be usable by the patient independently of a health care professional (e.g., orally available). In the case of MV, antivirals require systemic biodistribution to reach all lymphoid tissues and epithelia. Furthermore, antivirals should have low production costs and could ideally be synthesized at multiple locations. A long shelf stability would be advantageous to guarantee sufficient supply in case of outbreaks.

When discussing MV treatment options, we should not forget that measles is a difficult disease to treat. To date, most licensed antivirals are used in chronic disease such as HIV or HCV, which have their own challenges but which give ample opportunities to initiate and monitor treatment. An acute disease with a long asymptomatic phase and an early initiation of long-term immunosuppression such as measles leaves a narrow treatment window. Moreover, despite rising measles cases in the Western world, antivirals would mostly make an impact on children in measles-endemic countries. However, clinical trials in a pediatric patient population have major challenges, and as vaccination is yet the best standard of care, acceptance of clinical trials in a population not refusing vaccination will be difficult. This leaves researchers with a restricted population for clinical studies. Once marketed, target groups in the Western world include non-vaccinated healthcare workers and immunosuppressed patients, household or school contacts and patients suffering from measles-related neurological symptoms. Given the limited market, the research and development of an MV-specific antiviral may not be cost-effective, urging us to invest in broad-spectrum antivirals and a combination of targeted therapies. The latter will additionally be of increasing interest to reduce individual drug doses and related adverse events, as well as the emergence of drug-resistant viruses. It remains to be investigated if combination therapy of remdesivir or polymerase inhibitors with fusion inhibitory peptides will be a potent post-exposure prophylaxis for measles.

## Figures and Tables

**Table 1:** Antivirals investigated in vitro for MV.

Antiviral Class	Examples Investigated for MV In Vitro
**Nucleoside analogs**	Ribavirin and ribavirin-derivatives and -analogs [33,34,35,36,37,38,39,40] Ring-expanded purine nucleosides [34] Remdesivir [41,42] Favipiravir (T-705) [43,44] Derivatives of R1479 [45] Pyrazofurin [36] 3-Deazaguanine and its carbocyclic analog [36,38,40] 6-Azauridine [36] 5’-nor carbocyclic adenosine analogues [46] L-dideoxy bicyclic nucleoside analogs [47]
**Non-nucleoside polymerase inhibitors**	AS136a [48,49] ERDRP-0519 [49,50,51,52] GHP-88309 [53]
**Fusion and entry inhibitors**	**Peptide inhibitors** Z-D- Phe-L-Phe-L-Gly (FIP) [54,55,56,57,58] HRC-based peptides: M1, M2, M3, M4, M1EK, M2EK, M3EK, M4EK [59] HRC-lipopeptides [60,61,62,63,64] FIP-lipopeptides [64] FIP-HRC-lipopeptides [64] **Nonpeptidic small molecules [65]** 16677 [66] 3G [63,67] AS-48 [56,57,58,65,66] OX-1-(variants) [57,68] AM-2 [68] AM-4 [57] shRNA [69]
**Fusion and entry inhibitors**	
**Host-directed compounds**	ZHAWOC9045 and ZHAWOC21026 [70] EMXV-001299 and EMXV-1400 [71] Benzimidazole (JMN3-003) [71]
**Plant extracts**	Brassinosteroids [72] Polyphenol-rich extracts from seaweed [73] Extracts of Zanthoxylum chalybeum [74] Fucoidan (brown alga) [75] Extracts from cajanus cajan [76] Extracts from podophyllum peltatum [77] Hot Water extracts [78] *Olinia rochetiana* (Olkirenyi) extracts [79] *Warburgia ugandensis* (Osokonoi) extracts [74,79]
**Disinfectant**	Chlorine dioxide and sodium hypochloride [80]
**Anti-mycoplasmal and anti-microbial peptides**	2´-Amino-2´-Deoxyribofuranosyl Adenine (2-AA) [81] Mucroporin (optimized/attenuated) [82]
**Interferons**	IFNα [37]
**Others**	Inhibitors of cholesterol synthesis (W-7, cerulenin, mevinolin, miconazole, ketoconazole) [55] Carbobenzoxy-(di-or tri-) peptides [83] PPMO (anti-mRNA) [84] Pyrazino-Pyrazine derivate [85] PCG [86] ViroSAL (emulsion of short-chain caprylic acid) [87] Isoprinosine (inosine derivate) [88]

**Table 2:** In vitro antiviral assays used to determine the efficacy of antivirals studied for MV.

Assay	Description	Cells	Virus Strains
**Virus-yield reduction assay**	Determination of MV neutralization by a (serially diluted) compound. Compound potency is evaluated by titration of supernatants generated during incubation of MV in the presence of the antiviral compound. Titration can be performed as plaque assay, end-point titration (TCID_50_) or HA assay.	Vero [55,65,66,68,72,77,88] Vero E6 [82] CV-1 [46] Vero-humanSLAM [44,48,49,50,52,53,57,65,66,87,99] LLCMK [86] HEL-R66 [81] CK [85] U937 [79] human PBMC [52]	**MV Edmonston(-derived)** Edmonston [46,65,81,82,85] rMV-Edm [48,50,66] rMV-Edm-GFP [44,68] **MV wild-type(-derived)** MV/Brazil/001/991 [72] MVi/Alaska.USA/16.00 [48,49,50,53,99] MVi/Ibadan.NIE/97/1 [48,50] MVi-Amsterdam.NET/49.97 [48] MVi/Maryland.USA/77 [50] MVi/Illinois.USA/46.02 [50] MVi/NewJersey.USA/94/1 [50,52] MVi/Illinois.USA/50.99 [50] MVi/Kansas.USA/43.00 [57,65] MV Hallé strain [55] Chicago-1 [46] culture adapted Chicago 1 [79] MV-Ibd, MV-JM77, MV-NJ, MV-III 99, MV-Vic/Aus, MV-Amster.NET, MV Gresik, MV-Alaska [66] rMV-IC323-eGFP [87] **Undefined MV** [53,77,86,88]
**Neutralization assay**	Determination of (complete) neutralization by a (serially diluted) compound. Alternatively: Fixed concentration of compound but serially diluted concentration of virus. Compound potency is evaluated by readout of CPE/syncytia-formation-inhibition. Inhibition can be visually (microscopically) evaluated, potentially supported by crystal violet staining or by staining of the monolayer with neutral red and evaluation of optical density (neutral red inhibition assay).	Vero [36,37,40,59,65,68,73,74,77,88] Vero E6 [38] CV-1 [34,46] Vero-humanSLAM [48,57,59,65,80,99] CHO/SLAM [84] Hep-2 [76,83] B95a [47] BSC-1 [47] CK [85]	**MV Edmonston(-derived)** Edmonston [46,47,59,65,73,80,83,84,85] rMV-Edm [48] rMV-Edm-GFP [68] Schwarz [74] **MV wild-type(-derived)** MVi/Kansas.USA/43.00 [57,65] MVi/Alaska.USA/16.00 [48,99] MVi-Amsterdam.NET/49.97 [48] MVi/Ibadan.NIE/97/1 [48] CC [38] WTFb [47] Berkeley/83, Ibadan/97, Chicago1/89 [84] TN1994, Halonen, Bil, X-1108, SA, CC, Chicago-1 [46] Suguyama [36] Hep-2 adapted attenuated MeV [76] **SSPE viruses** Yamagata-1 [36,37,59] Niigata-1 [36] Kitaken-1 [36] **Undefined MV** [34,40,77,88]
**Antigen reduction assay**	Determination of (complete) neutralization by a (serially diluted) compound. Compound potency is evaluated by staining of the monolayer with an anti-MV antibody and evaluation of MV antigen expression.	Vero [33,35] HeLa [41] HEL-R66 [81]	**MV Edmonston(-derived)** Edmonston [33,35,41,81] **Adapted MV** CAM/RB [33] **SSPE viruses** Hallé [35] Mantooth [35] McClellan [35]
**Reporter assay**	Determination of (complete) neutralization by a (serially diluted) compound. Compound potency is evaluated by expression of a reporter protein, i.e., fluorescent protein or luciferase. Quantification of the reporter expression indicates potency of the compound.	Vero [68] VeroE6 [42,53] Vero-humanSLAM [44,51,53,60] Vero-dogSLAM [70] CHO/SLAM [56] CHO/CD46 [56] CHO/PVRL4 [56] HeLa [41] NCI-H358 [42,45] HSAEC1-KT [42] NT2 [69] HEK293T [69]	**MV Edmonston-derived** rMV-Edm-(E)GFP [41,42,44,45,56,68] rMV-Moraten-Luciferase [70] rMV-NanolucPEST [51,53] **MV wild-type-derived** rMV-IC323-eGFP [56,60] rMV-IC323-Luciferase [70] **Adapted MV-derived** rMVHcRed-CAMH [69] rMV-EGFP-CAMH [69]
**Plaque assay**	(Serially diluted) compounds added to overlay medium. After fixation, plaques are visualized with crystal violet, neutral red or fluorescently (reporter virus or immunostaining) and used to evaluate compound potency.	Vero [35,39,43,59,63,74,78,79] Vero E6 [82] Vero-humanSLAM [43,60,61,63] Hep-2 [83] HEL-R66 [81]	**MV Edmonston(-derived)** Edmonston [39,43,74,79,81,82,83] **MV wild-type-derived** Tanabe strain [78] Sugiyama [39] Toyoshima [39] rMV-IC323-eGFP [63] MV G954 [60,61] **SSPE viruses** Yamagata-1 [39,43,59] Hallé [35]
**(Chemiluminescent) complementation-based assay**	Cells transiently transfected with a MV receptor (and often one subunit of a reporter) are incubated with cells co-expressing MV glycoproteins H and F (and the other reporter subunit). The fusion process is evaluated in presence of compounds by read-out of reporter expression (i.e., chemiluminescence) or by microscopical analysis of syncytia formation.	Vero [56,67,68,99] Vero-humanSLAM [56,57,67] Vero-PVRL4 [56] HEK293 [56] HEK293T [61,63,64] HEK293T/SLAM [60,61,62,63,64] HEK293T/nectin-4 [62,63,64]	**MV Edmonston-derived** MV-Edm H, F [56,67,68] **MV wild-type-derived** MV-Kansas H/F [57] MV-G954 H/F [61] MV-IC323 H/F [60,62,63,64] **Undefined MV-derived** MV H/F [99]
**Replicon assay**	Evaluates compounds for their effect on the polymerase unit. In the assay a minigenome composed of MV-N, -P and -L and a reporter are used instead of live virus. The reporter can be CAT, a fluorescent or a luciferase protein. Quantification of the reporter evaluates the potency of the antiviral compound.	BHK/sr/T7 [84] BHK-T [48] BSR T7/5 [51,53,66,70] HEK293T [69]	**MV Edmonston-derived minigenomes** [48,51,53,66,69,70,84]
**RT-PCR**	Determination of neutralization/inhibition by a (serially diluted) compound. Compound potency is evaluated by detecting MV-genomes by RT-PCR.	Vero [48,73] Vero-humanSLAM [51,53]	**MV Edmonston(-derived)** Edmonston [73] rMV-Edm [48] **MV wild-type-derived** rMV-Anc (from MVi/Alaska.USA/16.00) [51,53]

TCID50: Tissue culture infectious dose 50; HA assay: hemagglutination assay; CPE: cytopathogenic effect; CAT: chloramphenicol acetyltransferase.
